# Protopanaxadiols Eliminate Behavioral Impairments and Mitochondrial Dysfunction in Parkinson’s Disease Mice Model

**DOI:** 10.1007/s11064-024-04132-w

**Published:** 2024-03-29

**Authors:** Jindong Zhao, Ji Wang, Kunying Zhao, Yuxiao Zhang, Weiyan Hu

**Affiliations:** 1 School of Pharmaceutical Science & Yunnan Key Laboratory of Pharmacology for Natural Products, Kunming Medical University, Kunming, 650500 People’s Republic of China; 2 College of Modern Biomedical Industry, Kunming Medical University, Kunming, 650500 People’s Republic of China; 3 School of Chinese Materia Medica &Yunnan Key Laboratory of Southern Medicine Utilization, Yunnan University of Chinese Medicine, Kunming, 650500 People’s Republic of China

**Keywords:** Protopanaxadiols, Behavioral impairments, Mitochondrial dysfunction, Parkinson’s disease

## Abstract

Currently, there are no effective therapies to cure Parkinson’s disease (PD), which is the second most common neurodegenerative disease primarily characterized by motor dysfunction and degeneration of dopaminergic neurons in the substantia nigra pars compacta (SNc). Protopanaxadiols (PPDs), including 20 (R)- protopanaxadiol (R-PPD) and 20 (S)- protopanaxadiol (S-PPD), are main metabolites of ginsenosides. The role of ginsenosides in neurodegenerative diseases has been thoroughly studied, however, it is unknown whether PPDs can attenuate behavioral deficits and dopaminergic neuron injury in PD model mice to date. Here, we administered PPDs to MPTP-induced PD model mice and monitored the effects on behavior and dopaminergic neurons to investigate the effects of R-PPD and S-PPD against PD. Our results showed that R-PPD and S-PPD (at a dose of 20 mg/kg, i.g.) treatment alleviated MPTP (30 mg/kg, i.p.) induced behavioral deficits. Besides, R-PPD and S-PPD protected MPP^+^-induced neuron injury and mitochondrial dysfunction, and reduced the abnormal expression of Cyt C, Bax, caspase-3 and Bcl-2. These findings demonstrate that R-PPD and S-PPD were potentially useful to ameliorate PD.

## Introduction

Parkinson’s disease (PD) is one of the most disabling diseases of the central nervous system (CNS) which affects over 10 million people worldwide per year [1–3]. Patients with PD suffer from both motor symptoms [4–6] (resting tremors, rigidity, bradykinesia, postural instability), and non-motor impairments [7] (autonomic dysfunction, hyposmia, etc.) which occur years before the onset of motor dysfunctions. Its neuropathological features include progressive degeneration of dopaminergic (DA) neurons, misfolding aggregation of α-synuclein, and the formation of Lewy bodies [8, 9]. Although the exact pathological mechanisms of PD remains unclear, mounting evidence reveals that cell apoptosis [10], mitochondrial dysfunction [11], and neuroinflammation [12, 13] may contribute to the onset and progression of this disease.

Mitochondrial dysfunction leads to oxidative stress and excessive release of reactive oxygen species (ROS), causing damage of the mitochondrial membrane [14]. The damage of mitochondrial membrane activates the apoptotic signaling pathway, resulting the activation of pro-apoptotic protein Bax and inhibition of anti-apoptotic protein Bcl-2 [15], inducing the release of Cyt C from the mitochondria, forming apoptotic bodies or initiating the activation of Caspase 9 [16, 17]. Once the executioner caspases are activated, they degrade cellular proteins through proteolysis, leading to a reduction in dopamine neurons and promoting the occurrence and development of PD.

Ginsenosides are the main bioactive compounds found in *Panax ginseng* and *Panax notoginseng*. A variety of ginsenosides (Rb1, Rb2, etc.) have been isolated and identified [18]. Due to its steroidal structure, ginsenosides have varying degrees of protective effects on the nervous system, digestive system, immune system, and more [19–21]. But its hydrophobicity leads to poor absorption through the intestine and low bioavailability [22, 23]. Diol-type ginsenosides have various biological activities, including anti-inflammatory, antioxidant, anti-apoptotic, and immune-regulating effects, making them natural neuroprotective agents, and have been shown to have potential in the treatment of PD [24–26]. Most ginsenosides can treat oxidative stress and inflammation by targeting Sirtuin 1 signaling pathway. Ginsenoside Rc reduces mitochondrial damage and apoptosis by inducing SIRT. In addition, ginsenoside Re inhibited neuroinflammation by down-regulating CAMK/MAPK/NF-κB signaling in microglia, thereby exerting a neuroprotective effect in LPS-induced microglia model. PPDs is the main metabolite of ginsenoside processed by human gut microbiota and has high bioavailability [27].Similar to diol-type ginsenosides, protopanaxadiols (PPDs, the metabolite of diol-type ginsenosides) also exhibit good anti-inflammatory [28, 29], antioxidant [22, 30, 31], and immune-regulating effects. It has also been found that PPDs have the potential to promote neurogenesis in vivo [27, 32]. However, the role of PPDs against PD have not been explored to date. Therefore, in this study we investigated the effects and possible mechanisms of PPDs administration on behavioral impairments and pathological changes using MPTP-induced PD model and MPP^+^-stimulated primary neurons.

## Materials and Methods

### Reagents

MPTP (M0896) and levodopa (D9628) were purchased from Sigma-Aldrich, 20R-PPD and 20S-PPD were obtained from Xililife Science (20210510). The following antibodies were purchased from Abcam: anti-TH (ab137869), anti-Cytochrome C (ab133504), anti-Bax (ab32503), anti-caspase-3 (ab184787), Goat Anti-Rabbit IgG & L (HRP) (ab6721), and Goat Anti-Mouse IgG H & L (HRP) (ab6789). Bcl-2 (AF6139) was purchased from Affinity. Lastly, anti-β-actin (A5441) was purchased from Sigma-Aldrich.

### Animals and Treatment

All C57BL/6 mice (male, weighing 22–25 g) were purchased from Department of Zoology & Yunnan Key Laboratory of Pharmacology for Natural Products, Kunming Medical University (Kunming, China), and housed in a room under a 12 h light/dark cycle with free access to food and water. Before experiments, all mice were kept for a 1-week acclimatization period.

**Fig. 1:**
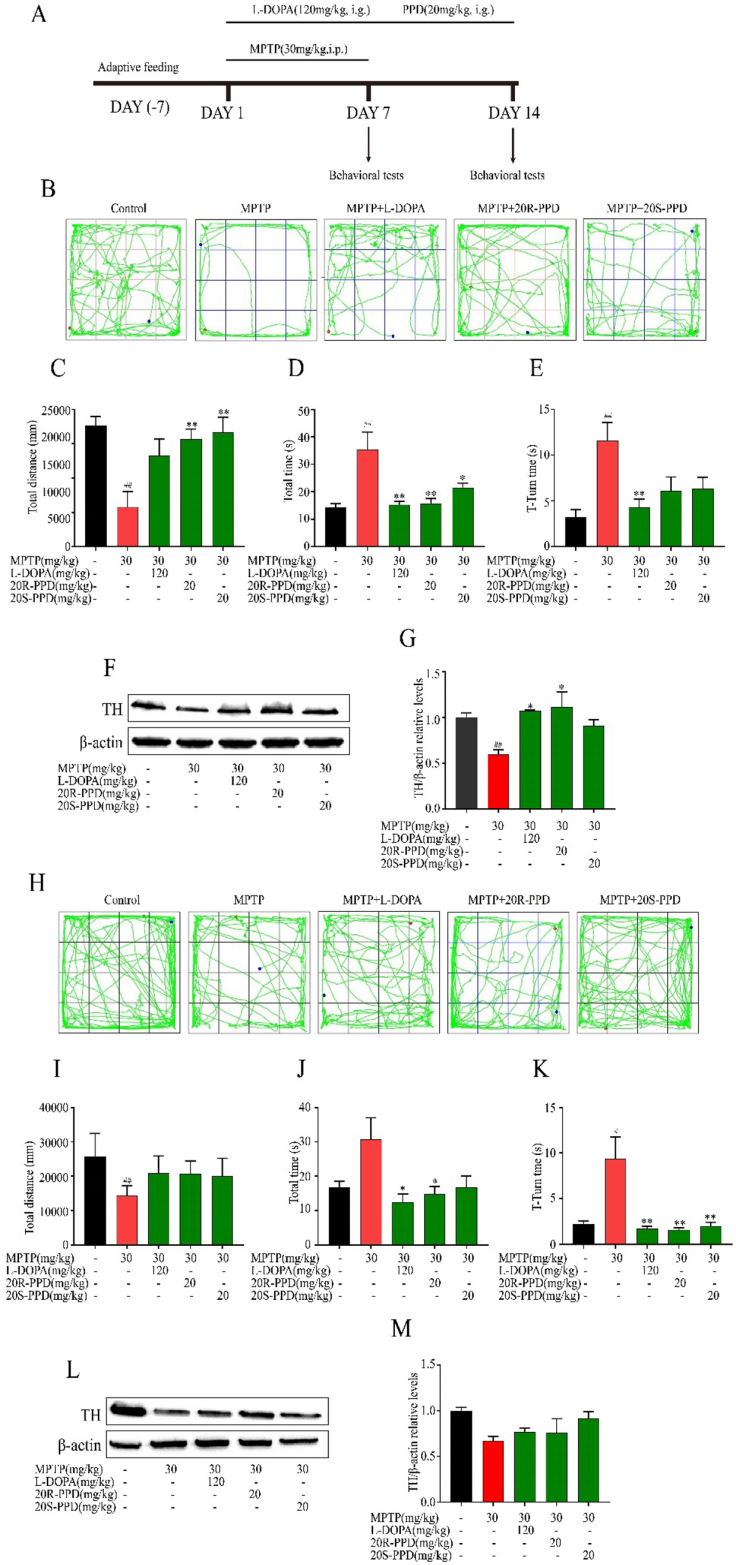
PPDs improve motor behavior and the reduction of TH protein in MPTP-treated mice. Behavioral parameters were measured on D 7 (n = 9) and D 14 to assess motor function in different treatment groups (n = 6). **A** Timeline of the experimental procedure in the MPTP-induced mouse PD model. **B** and **C** Day 7 open field test. **D** and **E** Day 7 pole test. **F** and **G** TH expression on Day 7 (n = 3). **H** and **I** Day 14 open field test. **J** and **K** Day 14 pole test. **L** and **M** TH expression on Day14 (n = 3). Quantified data are normalized to the control group (the control group value is equal to 1). Data are expressed as means ± S.E.M, (n = 6). Statistical significance was determined by one-way ANOVA followed by Tukey’s post hoc analysis where ^#^P < 0.05, ^##^P < 0.01 represents control vs. MPTP group, *P < 0.05, **P < 0.01 represents MPTP vs. MPTP + protopanaxadiols or L-DOPA

To evaluate the effects of 20R-PPD and 20S-PPD on MPTP-induced PD mouse model [33, 34], mice were randomly divided into the following five groups: control group (saline treated), model group (MPTP-treated, 30 mg/kg, i.p.), 20R-PPD group (MPTP and 20R-PPD treated), 20S-PPD group (MPTP and 20S-PPD treated), levodopa group (MPTP and levodopa treated). MPTP-HCL was dissolved in normal saline (0.9%) solution and injected intraperitoneally for 7 consecutive days. PPDs and levodopa were dissolved in 0.9% saline and administered by gavage for 14 days at doses of 20 mg/kg and 120 mg/kg respectively (Fig. 1A).

### Behavioral Testing

#### Open Field Test (OFT)

The open field test is widely used to assess spontaneous locomotor activity in mice. The Open field box consists of a plastic box of 50 cm × 50 cm × 50 cm. Mice are placed in the center of the open field and behavioral activities were videotaped for 5 min [35].

#### Pole Climbing Test

The pole-climbing experiment is a classic method for evaluating the coordination ability of mice. Mice were placed head up on a pole (length: 50 cm, and radius: 0.5 cm). The time which mice head turned (T-turn) and the total time from the top to the bottom of the pole was recorded.

### Cell Culture Treatments

The midbrain of the fetuses was removed from the pregnant mice at 18 days of gestation under anesthesia. After the blood membranes and blood vessels of the midbrain tissue were mechanically stripped off, the cell suspension was obtained by adding 0.25% trypsin for 15 min. The neurons were mixed with culture medium, evenly planted in 96-well or 6-well plate in Neurobasal medium supplemented with 50 × B27 supplement for a final concentration of 1 × . Then, neurons were transferred to the cell culture incubator (37 °C, 5% CO_2_) for further culture, and the medium was changed the next day. Neuronal cells mature at 7–8 days. Dopaminergic neurons were examined by immunocytochemical staining using antibodies against the neuronal marker β-III-tubulin and the dopaminergic marker tyrosine hydroxylase (TH).

### CCK-8 Assay of Cell Viability

The effect of PPDs on neurons viability was detected using a cell counting kit (CCK8) (C8022, Adamas life). Briefly [36], primary neurons were seeded in 96 well plates. After exposure to different concentrations of PPDs (5, 10, 20 μmol/L) and MPP^+^ (30 μmol/L) for 24 h, 10 μL of CCK8 solution was added to each well and incubated for 2 h. Finally, the absorbance was detected at 450 nm.

### Cell ROS

The intracellular ROS levels were measured using the DCFH-DA assay kit [37]. Cells pretreated with (or without) PPDs (10 μmol/L) were primed with MPP^+^ (30 μmol/L, 24 h). 10 μmol/L of DCFH-DA reagent was added and incubated at 37 °C for 20 min. After that, cells were washed three times with PBS. The fluorescence intensity was observed under a fluorescence microscope and images were captured.

### Mitochondrial Membrane Potential

The JC-1 mitochondrial membrane potential fluorescence probe was used to detect changes in mitochondrial membrane potential. After 24 h of PPDs (10 μmol/L) and MPP^+^ (30 μmol/L) treatment, 10 µg/mL JC-1 working solution was added and incubated for 20 min at 37 °C, followed by washing with PBS for three times. The fluorescence intensity was observed under a fluorescence microscope and photographed or the absorbance was detected by a fluorescent microplate reader.

### Western Blot Analysis

The proteins from cell or tissue samples were separated by SDS-PAGE polyacrylamide gel and transferred onto a polyvinylidene difluoride (PVDF) membrane. The membrane was blocked in 5% skim milk for 2 h, followed by overnight incubation with protein-specific antibodies on a shaker at 4 °C. After incubation with HRP-conjugated secondary antibodies for 2 h, chemiluminescent substrate was evenly added onto the PVDF membrane containing the target protein, and chemiluminescent imaging was performed using a chemiluminescent imaging system.

### Data Statistics and Analysis

The data were tested for homogeneity of variance and normal distribution, difference between groups was analyzed with one-way ANOVA followed by a Tukey’s post hoc test. The data results were presented as mean ± standard error of the mean (Mean ± SEM). A P-value of < 0.05 is considered statistically significant.

## Results

### PPDs Ameliorate Motor Deficits and TH Reduction in MPTP-Administered miCe

Behavioral tests were conducted on Day 7 and Day 14 to assess the motor symptoms of the mice. The open field test was used to evaluate the mice’s spontaneous activity and motor impairment. Compared to the control group, MPTP mice exhibited a significant decrease in the distance traveled in the open field on Day7 and 14 (Fig. 1B and C, H–I). The pole test was done to assess motor coordination. Compared to the control group, MPTP mice spent more time for both t-turn and t-total on Day 7 (Fig. 1D and E) and 14 (Fig. 1J and K), indicating severe impairment in motor coordination. Compared to MPTP mice, PPDs at a dose of 20 mg/kg body weight significantly alleviated motor impairment of MPTP-induced PD model mice in the pole climbing and open field tests on Day 7 and Day 14.

Tyrosine hydroxylase (TH) is a key enzyme in the dopamine (DA) biosynthetic pathway. PD is a neurodegenerative disorder caused by a severe deficiency of DA in the substantia nigra and striatum. Therefore, TH plays an important role in the biosynthesis of dopamine, and the changes of TH content are closely related to the occurrence and development of PD. We detected the expression levels of TH protein by western blot. The results showed that the expression of TH protein was reduced in MPTP-treated mice, while PPDs treatment significantly increased TH expression (Fig. 1F and G, L and M).

### PPDs Attenuate MPP^+^-Induced Primary Neurons Injury and Mitochondrial Deficits

**Fig. 2:**
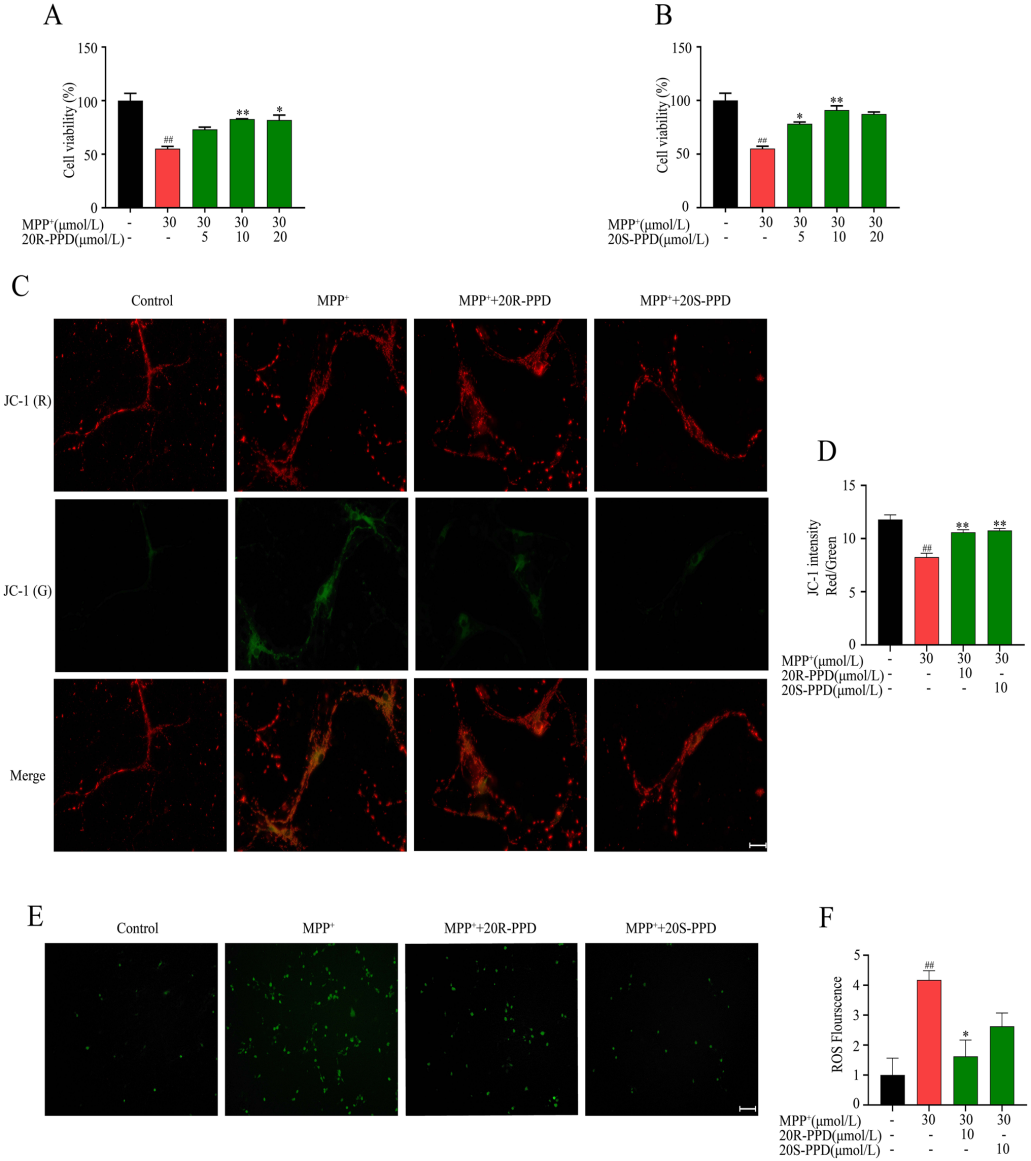
The effects of PPDs on MPP^+^-induced neuronal injury and mitochondrial deficits. A R-PPD. B S-PPD. **A** and **B** Primary neurons were pretreated with R-PPD and S-PPD for 2 h, followed by MPP^+^ treatment for 24 h, and the cell viability was measured by CCK8. **C** Mitochondrial membrane potential was measured by JC-1. In healthy cells, JC-1 monomers aggregate to form polymers, and mitochondria exhibit intense red fluorescence. JC-1 is present as a monomer in apoptotic or necrotic cells, and mitochondria are strongly green fluorescence. Scale bars, 25 μm. **D** The absorbance of JC-1 was detected by fluorescent microplate reader. The ratio of red fluorescence signal to green fluorescence signal was calculated to judge the health of mitochondrial membrane potential. **E **The DCFH-DA method measures intracellular reactive oxygen species (ROS) accumulation and is monitored by fluorescence microscopy. Scale bars, 200 μm. **F** ROS fluorescence intensity was analyzed and quantified. Quantified data are normalized to the control group (the control group value is equal to 1). Statistical significance was determined by one-way ANOVA followed by Tukey’s post hoc analysis where ^#^P < 0.05, ^##^P < 0.01 represents control vs. MPP^+^ group, *P < 0.05, **P < 0.01 represents MPP^+^ vs. MPP^+^  + protopanaxadiols

To evaluate the effects of PPDs on the survival of primary neurons, primary neurons were pretreated with different concentrations of PPDs for 2 h, followed by treatment with MPP^+^ (30 μmol/L) for 24 h, and a CCK-8 assay was determined neuron viability. As shown in 2A and B, compared to the control group, the neuron viability was significantly lower in the MPP^+^ group. While, in the 20R-PPD and 20S-PPD groups, the cell viability was significantly higher than in the MPTP group (Fig. 2A andB). These results indicated that PPDs protected MPTP-induced neuron injury.

In order to check the effects of PPDs on mitochondrial deficits**,** mitochondrial membrane potential and cytoplasmic ROS were measured. As shown in Fig. 2C–F, MPP^+^ treatment impaired mitochondrial membrane potential and induced ROS production, whereas PPDs treatment restored mitochondrial membrane potential and inhibited the increase in cellular ROS levels.

### PPDs Administration Rescues MPTP-Induced the Abnormal Expression of Mitochondria-Mediated Apoptosis Proteins

**Fig. 3:**
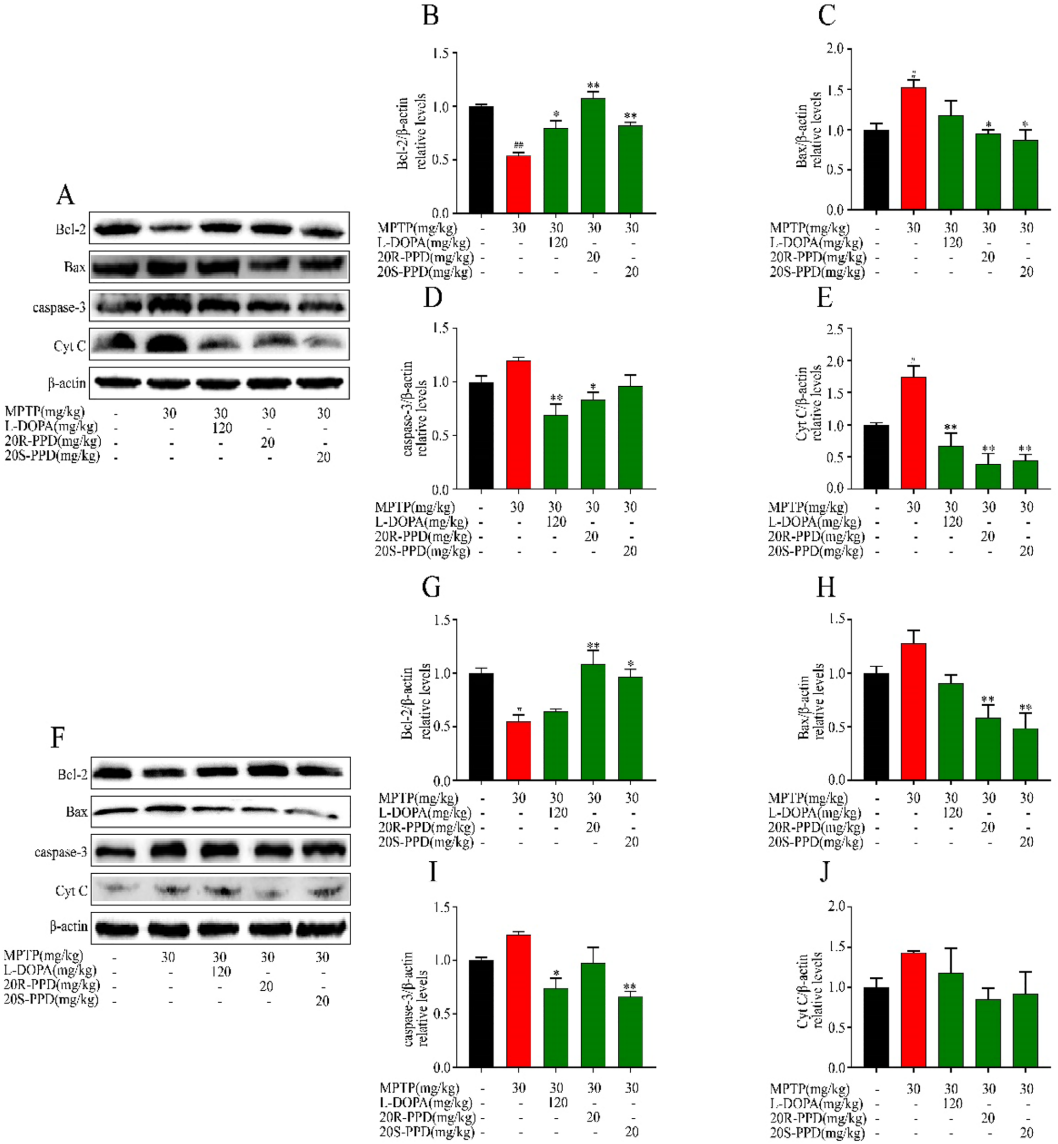
Effect of PPDs on MPTP-induced mitochondria-mediated apoptosis proteins. **A**–**E** The expressions of Bcl-2, Bax, caspase-3 and Cyt C were detected by western blot on Day7. **F**–**J** The expressions of Bcl-2, Bax, caspase-3 and Cyt C were detected by western blot on Day14. Quantified data are normalized to the control group (the control group value is equal to 1). Data are expressed as means ± S.E.M, (n = 3). Statistical significance was determined by one-way ANOVA followed by Tukey’s post hoc analysis where ^#^P < 0.05, ^##^P < 0.01 represents control vs. MPTP group, *P < 0.05, **P < 0.01 represents MPTP vs. MPTP + protopanaxadiols or Levodopa

Mitochondrial pro-apoptotic factor release into the cytoplasm plays a crucial role in mediating apoptosis in the occurrence and development of PD. In order to investigate the effects of PPDs on apoptosis, apoptosis-related proteins were extracted for detection. Compared to the control group, the expression of Cyt C increased in MPTP mice, and the expression of apoptosis-related proteins Bax and caspase-3 significantly increased, while the expression of anti-apoptotic protein Bcl-2 significantly decreased. While, after PPDs treatment, the expression of anti-apoptotic protein Bcl-2 increase, and the expression of apoptosis proteins Bax and caspase-3 increase (Fig. 3A–J). In conclusion, our results suggest that PPDs inhibit mitochondria-mediated cell apoptosis.

### PPDs Rescue MPP^+^-Induced the Abnormal Expression of Proteins Related to Mitochondria-Mediated Apoptosis

**Fig. 4:**
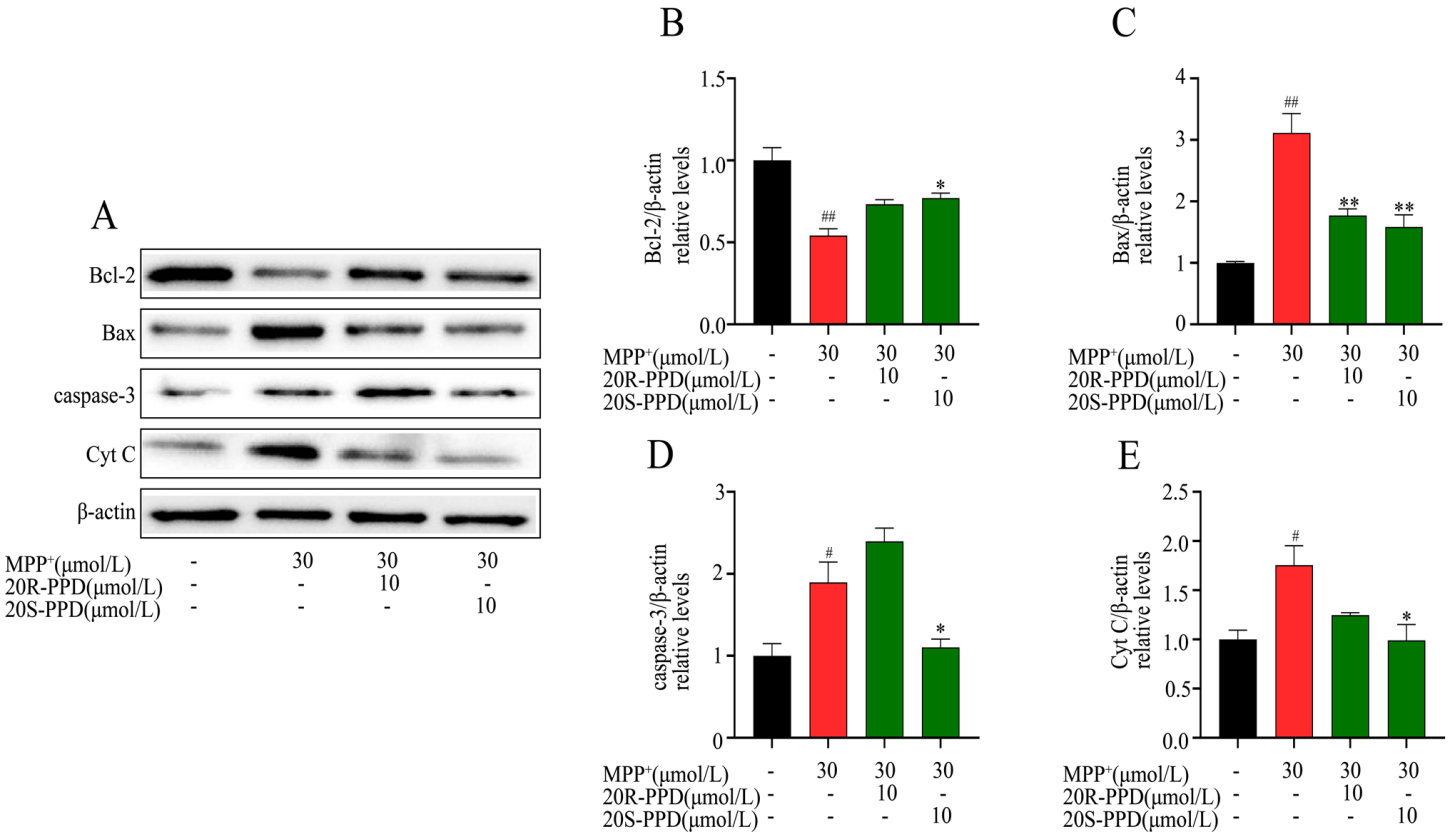
Effect of PPDs on MPP^+^-induced mitochondria-mediated apoptosis proteins. **A**–**E** The expression of apoptosis proteins Bcl-2, caspase-3, Bax and Cyt C in primary neurons was detected by western blot. Quantified data are normalized to the control group (the control group value is equal to 1). Data are expressed as means ± S.E.M, (n = 3). Statistical significance was determined by one-way ANOVA followed by Tukey’s post hoc analysis where ^#^P < 0.05, ^##^P < 0.01 represents control vs. MPP^+^ group, *P < 0.05, **P < 0.01 represents MPP^+^ vs. MPP^+^  + protopanaxadiols

In order to support the experimental results in MPTP-induced mice model, the MPP^+^ -induced primary neuronal model was analyzed. The results of the experiment showed that after treatment with MPP^+^, the levels of Cyt C significantly increased, as well as the levels of apoptotic proteins Bax and caspase-3, while the expression of anti-apoptotic protein Bcl-2 decreased. However, when we treated the cells with PPD, these changes were reversed (Fig. 4A–E).

## Discussion

According to epidemiological studies, apoptosis and the development of PD are associated with impaired mitochondrial complex I activity and REDOX state imbalance [3, 38]. Although there are many kinds of drugs used in the clinical treatment of PD, there is no drug that can completely cure PD [39]. In recent years, with the increasing attention paid to traditional medicine and natural medicine, natural medicine for the treatment of diseases has received considerable attention. Therefore, the present study aimed to investigate the protective effect of protopanaxadiols (metabolites of ginsenoside) on motor deficits and mitochondrial dysfunction in MPTP-induced PD mice.

The main pathological manifestations of PD are movement disorders such as resting tremor, myotonia, bradykinesia, postural balance disorder and so on [40–42]. To investigate the effect of Protopanaxadiols in vivo, we induced a subacute PD model in mice by administering MPTP for 7 consecutive days, which resulted in significant motor impairment and reduced expression of TH protein [33–35]. In addition, MPTP is converted to 1-methyl-4-phenylpyridine (MPP^+^) in glial cells, which blocks complex I of the electron transport chain, reduces ATP production and increases ROS production, resulting in mitochondrial dysfunction [43, 44]. When mitochondrial function is impaired, a series of signaling molecules is released, among them Cyt C and apoptosis-inducing factors [21, 45], which can activate the apoptotic pathway, which is consistent with previously reported inhibition [46–48]. Treatment with Protopanaxadiols ameliorated MPTP-induced dyskinesia, improved motor coordination and spontaneous movements, and increased TH protein expression in PD mice. Meanwhile, MPTP-induced increase of Bax, caspase-3 and Cyt C protein expression and decrease of Bcl-2 protein expression were reversed.

The mitochondria are critical regulators of function and survival within neurons in the brain, playing a vital role in maintaining neuronal energy balance and preventing apoptosis [49–51]. In pathological autopsies of PD animal models and PD patients, a large number of enlarged and damaged mitochondria were found in neurons, indicating that dysfunctional mitochondria were accumulated in the body [52, 53]. Existing studies have shown that the increase of ROS and the decrease of ATP caused by mitochondrial dysfunction promote oxidative stress response, aging and neurodegenerative changes, which promote the occurrence and development of PD [54, 55]. MPP^+^ inhibits the function of respiratory complex I, interferes with the mitochondrial electron transport chain, and eventually leads to the decrease of mitochondrial membrane potential, the increase of ROS, and mitochondrial damage [55, 56]. We observed that PPDs treatment restored mitochondrial membrane potential and reduced ROS accumulation, suggesting that PPDs could rescue mitochondrial dysfunction. In addition, the in vitro findings were consistent with those in vivo, where PPDs inhibited mitochondria-mediated apoptosis and reversed the MPP^+^-induced abnormal increase in Bax, caspase-3, and Cyt C protein expression and abnormal decrease in Bcl-2 protein expression.

In Parkinson’s disease, levodopa, the precursor of dopamine, is a key component in the treatment of PD. It is often used to treat motor symptoms of PD. Studies have shown that levodopa therapy increased dopamine release and alleviated motor symptoms, it did not appear to affect the progression of the disease itself [57]. In our study, we observed that levodopa abolished the effect of MPTP on apoptotic factors, which contradicts the literature reports on the neuroprotective effects of levodopa. A possible explanation may be related to the complexity of PD pathogenesis. Existing studies found that Levodopa increased the activity of Mn-SOD and mitochondrial complex I when administered simultaneously with MPTP [58], this is consistent with previous reports [59]. In addition, Levodopa can upregulate neuronal growth and repair processes [60]. In the Rotenone-induced PD model, administration of Levodopa significantly reduced the activity levels of Bax and caspase-3 and increased the level of Bcl-2 [61, 62]. Mitochondria play an important role in regulating apoptosis and the pathogenesis of PD. Bax promotes apoptosis by inducing mitochondrial membrane depolarization and Cyt C release, while Bcl-2 inhibits apoptosis by preventing mitochondrial membrane depolarization [63]. Thus, while levodopa primarily treats motor symptoms through dopamine supplementation, the disease involves a complex interplay of multiple molecular and cellular pathways in addition to dopamine modulation, demonstrating the complexity of PD and the need for a comprehensive approach when investigating its mechanisms and potential treatments.

Therefore, our research demonstrates that PPDs improve motor dysfunction and restore mitochondrial dysfunction in PD model mice, thus suggesting potential use of PPDs for PD therapy.

## Data Availability

The data that support the findings of this study are available from the corresponding author upon reasonable request.
